# Some Salient Points in Canine Practice1Presented for consideration at the meeting of the Minnesota State Veterinary Medical Association, Owatonna, July 14, 1897,

**Published:** 1897-09

**Authors:** Walter Amos

**Affiliations:** Owatonna, Minn


### SOME SALIENT POINTS IN CANINE PRACTICE.[1]

By Walter Amos, V.S.,
OWATONNA, MINN,

With none of our friends and helpers among the lower animals would we part so reluctantly as with the dog. No speechless associate of man has ever so entwined itself around the very roots of our domestic life as the dog; none has won so much admiration, confidence, and affection; none has appealed to so large a number of mankind of every condition, age, and sex. It will, therefore, be conceded that so noble, so intelligent, and so faithful an animal as the dog is entitled to the most complete understanding and the best usage of which we are capable.

The professional treatment of the dog in disease naturally falls to the veterinarian; but, inasmuch as the dog is very different in his nature from the horse and other herbivora which engage the chief attention of the veterinary profession, it follows that if the dog is to be treated on a rational basis, he must be made the subject of special study by the veterinarian.

A knowledge of equine medicine goes but a little way to qualify a man to treat the dog successfully, and the sooner this is recognized by the profession of comparative medicine, the better it will be for both the profession and our canine friends. If the veterinarian hopes largely to acquire the confidence of the public as regards dogs, he must show not only that he has a grasp of medicine as medicine, but a special knowledge of the nature, varieties, and peculiarities of dogs. The dog must be understood in health, before his ailments can be appreciated and treated, and the more intelligent body of breeders and owners of dogs thoroughly recognize this. To successfully treat a dog in sickness, and for the veterinarian to be proficient, he must study the dog in health. By this course the individual under treatment is known, and thus acts as a mariner's compass as to the diagnosticating of the disease. The principle will bear repeating: Try, as much as possible, to become conversant with the habits and actions of the dog in health, and the diagnosis of disease will be greatly simplified.

We have now to consider those deviations from the normal which constitute what is termed "disease."

Disease is nothing more than an altered function, a more or less

[1] Presented for consideration at the meeting of the Minnesota State Veterinary Medical Association, Owatonna, July 14, 1897, departure from the natural condition; hence to know what is the natural condition of an animal is the first requisite for the understanding of disease. We must always have a standard of comparison. Scientific medicine is impossible without scientific pathology, or knowledge of altered function, and this, again, is dependent on a sound physiology or knowledge of the normal behavior of the body. Treatment is based on both as well as a knowledge of causation.

The most useful instrument in diagnosticating disease in a dog is the thermometer, and here is where the value of being thoroughly acquainted with dogs and their temperament takes place, or becomes valuable, for we must recognize differences not only for breed, age, sex, etc., but also for each individual. From personal observation and experiments made in taking the temperature of dogs of all kinds, from the great Dane and mastiff to the rat-terrier and lap-dog, as well as from information obtained from reliable authors and other sources, we find that the temperature of the dog very rarely falls below 100°, and it is also important to note that it may rise to 102° or even 102.5°, so that a conclusion that fever exists cannot be made on a reading of 102.5°, especially in a puppy, in which the temperature may naturally be higher than in an adult dog, and is liable to oscillate, as in human infants, very rapidly. But temperature under 99° or over 103° should arouse suspicion of disease, and even a temperature of 102·6°, if constant, cannot be normal.

The pulse, as indicating the rapidity and character of the heart-beats and the condition of the arteries, conveys to the experienced a world of information. It may be conveniently taken in the groin of the dog, but often it will be necessary, especially in the case of small dogs, to attempt to get at the state of the heart directly, which can readily be done in the dog by feeling the organ through the chest-wall. As the dog is a very excitable animal, he must be quieted and soothed as much as possible when the pulse is being taken, or the heart examined, especially by a stranger. In all cases it must be ascertained that the pulse is not merely transiently affected as the result of temporary excitement from the very process of examination or otherwise. Also the variations natural to the different position of the body are not to be forgotten.

The pulse at birth is very rapid—130 to 160; for the first three months, 120 to 140; at from the sixth to the ninth month, 90 to 110; after one year, 70 to 90. It must be understood that these are only rough estimates; so wide are the variations with age, sex, breed, position, temperament, etc. A merely rapid pulse, with no elevation of temperature or other unfavorable symptoms, is not of great significance usually. It is to be borne in mind, too, that when an adult dog is quietly sleeping the pulse may be very slow, even to 40 beats. I also wish to draw special attention to a feature of the pulse of the dog to which reference is seldom made—that is, with each expiration the pulse is slower and stronger and beats uneven —a condition of things which, if in man or any other animal, would be certain signs of disease in a large proportion of cases, and a dog that shows the phenomena in question decidedly is never a puppy, and they are never absent in a mature dog, so that these constitute in some measure an indication of age. By the inexperienced these peculiarities might readily be mistaken for abnormalities of the heart. The ratio of the respiration and pulse in the dog is about the same as in man—that is, about one to four when the dog is not heated or excited by overexertion.

It can readily be seen from the foregoing that the student of canine medicine should associate as much as possible with the dog to acquire the desired familiarity. The idea that a student of medicine can get all the knowledge of the dog that is required from seeing sick animals as they may be brought to an infirmary, though widespread, has not proved correct, and perhaps explains in no small degree that lack of confidence in veterinary surgeons as regards the dog, which is certainly prevalent, if not well founded. Departure from the normal can only be adequately appreciated by him who knows the normal (healthy) dog.

The physical examination of the dog is easy in itself, but may be troublesome if the animal is restive or fractious. I have heard even veterinary surgeons say that the dog should be taken out of sight of its master, quickly thrown down and so handled that he will be taken by surprise and offer no resistance. Remember a dog is a being of great sensitiveness, of strong likes and dislikes, and has a remarkable instinct and memory. A dog may be so treated that it will be almost impossible for the same person ever to succeed a second time in examining him or giving medicine. Moreover, if a dog resists it is impossible to form a correct judgment, as his functions are disturbed by that resistance. If a dog is treated so that he shall see that no harm is meant him, he will usually quietly submit according to his natural amiability, a little rubbing of the head, a few soothing words, a gradual approach toward the real object may occupy a few minutes at first, but save indefinitely in the end. Now and then there are exceptions; but in my own experience they are very rare. Therefore, in making an examination of the dog it is most important to do it in such a way as will render it easy of accomplishment on the next occasion; unless this be carried out so that it is at least not disagreeable to the animal, or as little so as possible, the trouble will increase on each repetition.

The closer observation and investigation we make the more we are led to see the similarity between the physical constitution of the canine race and that of man, also the similarity in the character of his diseases and the way in which medicines and other remedies react on him. It is quite impossible to treat the dog successfully on the same principles as the horse, ox, etc., while with comparatively few exceptions human medicine is directly applicable to the dog. As the treatment of the dog falls to the veterinarian and not to the human physician, it is all the more necessary that a special study should be made of the dog, taking human, rather than equine medicine and doses, as the standard of comparison ; hence in treating the dog we have to use the same or similar food-stuffs, similar medicines, in similar doses, and the same external and internal treatment generally with man.

A very few drugs are known to be required in larger doses for the dog than for man—aloes ; but this medicine alone is not a good remedy for either dog or man. Dogs are peculiarly liable to be salivated and even poisoned by the use of calomel, or mercury, in any form, so that great care must be taken to see that it is administered in very small doses ($\frac{1}{4}$ to 3 grains), and speedily removed from the system by a saline or other aperient. Turpentine is another drug dangerous to the dog, but the same applies to man. It is also important to note that opium or morphine can be tolerated to an almost unlimited extent. A large dose of opium nauseates a dog profoundly, but is not at all likely to poison him.

A rule for the dose of any medicine given to the dog, based on that given to the horse or any other animal, is extremely difficult to lay down. In general it may be said that for the largest dog (St. Bernard or mastiff) the dose is the same exactly as for a full-grown man. For a dog of, say, forty pounds, a dose about the same as for a fourteen-year-old boy, or two-thirds that of a man. In the case of small matured dogs, such as terriers, spaniels, pugs, etc., one-third to one-half the latter dose will be near the mark. In the case of puppies two months old or under, the less medicine given the better ; very few and small doses. For constipation in suckling puppies treat through the mother.

Methods of administering medicine are of similar importance to the method of examination, before given in this paper, because of the difficulties that arise if the animal becomes unmanageable or objects seriously to the treatment. Harshness with dogs is so radically opposed to their nature that it in every way defeats the end in view.

Each practitioner has his own methods of giving medicine. I will here give a few methods:

1. Get the dog backed into a corner; press the lips against the teeth; when the mouth is open, pass the medicine far back, rapidly close the mouth, and wait for the dog to swallow, covering the nostrils, if necessary, to compel him to do so.

2. Insert the mouth of a small-necked bottle containing the medicine in the pouch between the angle of the lips, pouring the liquid back.

3. Pouring medicine down, if liquid, from a spoon.

4. Giving pills in meat.

5. Attaching a cloth to the upper and lower jaw to hold them open in the case of dogs large and hard to manage.

6. Keep the head up after administering, to prevent vomiting.

While the above methods may be useful as guides, and successful or necessary in some cases, the nature of the dog and the amount of experience one has must greatly determine the method. If possible, it is well that a dog should not know that he is getting medicine at all, so that if the latter can be given in small pieces of meat it is most desirable. Modern pharmacy has greatly aided the canine practitioner in the manufacture of pills and tablets and capsules, thus making medicine easy of administration, as a dog's stomach will not tolerate nauseous draughts. So many drugs are now put up in useful combinations for man's use that we can avail ourselves of a large field for selection. Gelatin capsules No. 0, No. 00 serve every purpose for dogs. It is often necessary to give castor-oil to dogs. This is done with greater ease if the spoon and oil are both heated, when it will run off the spoon and down the dog's throat easier. Quickness in the administration of medicines is of great importance.

My own way of giving medicine to a dog is: Never use fluids that must be poured down if possible to avoid it. Get the dog against a wall, a corner preferred, between the knees, press on the lips just enough to cause the mouth to open, then insert the gelatin capsule, moistened with saliva, far back on the tongue, and giving it a gentle push with the fingers, suddenly close the dog's mouth, when he usually swallows, unaware almost of what has happened.

Then pat him on the head, say an encouraging word, and he has no worse opinion of his physician than before.

Hypodermics are usually not advisable in the treatment of the dog, only when the stomach will not tolerate medicine.

In all dealings with dogs the motto should be: Decision, rapid action, and gentleness.

